# Automated information extraction model enhancing traditional Chinese medicine RCT evidence extraction (Evi-BERT): algorithm development and validation

**DOI:** 10.3389/frai.2024.1454945

**Published:** 2024-08-15

**Authors:** Yizhen Li, Zhongzhi Luan, Yixing Liu, Heyuan Liu, Jiaxing Qi, Dongran Han

**Affiliations:** ^1^School of Computer Science and Engineering, Beihang University, Beijing, China; ^2^School of Management, Beijing University of Chinese Medicine, Beijing, China; ^3^School of Life and Science, Beijing University of Chinese Medicine, Beijing, China

**Keywords:** online clinical literature, Evi-BERT, traditional Chinese medicine, RCT, knowledge engineer

## Abstract

**Background:**

In the field of evidence-based medicine, randomized controlled trials (RCTs) are of critical importance for writing clinical guidelines and providing guidance to practicing physicians. Currently, RCTs rely heavily on manual extraction, but this method has data breadth limitations and is less efficient.

**Objectives:**

To expand the breadth of data and improve the efficiency of obtaining clinical evidence, here, we introduce an automated information extraction model for traditional Chinese medicine (TCM) RCT evidence extraction.

**Methods:**

We adopt the Evidence-Bidirectional Encoder Representation from Transformers (Evi-BERT) for automated information extraction, which is combined with rule extraction. Eleven disease types and 48,523 research articles from the China National Knowledge Infrastructure (CNKI), WanFang Data, and VIP databases were selected as the data source for extraction. We then constructed a manually annotated dataset of TCM clinical literature to train the model, including ten evidence elements and 24,244 datapoints. We chose two models, BERT-CRF and BiLSTM-CRF, as the baseline, and compared the training effects with Evi-BERT and Evi-BERT combined with rule expression (RE).

**Results:**

We found that Evi-BERT combined with RE achieved the best performance (precision score = 0.926, Recall = 0.952, F1 score = 0.938) and had the best robustness. We totally summarized 113 pieces of rule datasets in the regulation extraction procedure. Our model dramatically expands the amount of data that can be searched and greatly improves efficiency without losing accuracy.

**Conclusion:**

Our work provided an intelligent approach to extracting clinical evidence for TCM RCT data. Our model can help physicians reduce the time spent reading journals and rapidly speed up the screening of clinical trial evidence to help generate accurate clinical reference guidelines. Additionally, we hope the structured clinical evidence and structured knowledge extracted from this study will help other researchers build large language models in TCM.

## Introduction

In evidence-based medicine, randomized control trial (RCT) results provide essential data for and add significant value to systematic reviews and meta-analysis, which aid in clinical decision-making and medicine guideline development (Stylianou et al., 2020). Physicians can construct semantic networks and knowledge graphs by extracting and analyzing RCT data (Kang et al., 2023; Malec et al., 2023). Traditional Chinese medicine (TCM) has a rich history of utility for over 2,000 years, and is a unique and vital branch of medicine. RCTs play a crucial role in elucidating the therapeutic effects of TCM (Liu et al., 2016, 2017; Tu et al., 2021). However, currently, clinical evidence extraction for RCTs is largely done manually and is subject to shortcomings in efficiency and breadth, and is likely vulnerable to subjective bias.

From knowledge engineering to machine learning (ML) and deep learning (DL) algorithms, Information extraction (IE) technology (Chen et al., 2021; Guo et al., 2024), which commonly solves sequence annotation tasks such as named entity recognition, has rapidly developed into natural language processing (NLP) (Foufi et al., 2019; Li Y. et al., 2022; Sugimoto et al., 2023). ML models, like Conditional Random Field (CRF) (Liu and El-Gohary, 2017), rely on statistical learning to achieve powerful results. DL models, like the long short-term memory (LSTM) network (Hochreiter and Schmidhuber, 1997) and bidirectional long short-term memory (Bi-LSTM) network (Graves and Schmidhuber, 2005), have the ability to incorporate contextual information and can be utilized independently or in tandem with other machine learning frameworks. The LSTM-CRF model architecture has emerged as a popular tool for named entity recognition, with applications ranging from automatic extraction of named entities in cyber threats (Kim et al., 2020) and legal texts (Xu and Hu, 2022) to Chinese word segmentation (Li et al., 2020). IE technology has been applied to many general-knowledge fields, such as commercial operations for user data capture, understanding emotional reasoning, and AI Question Answer sequences (Maruf et al., 2021; Zhang et al., 2022; Zirikly et al., 2022).

End-to-end IE methods have been shown to achieve excellent results in sequence labeling tasks such as attention mechanisms and transfer learning models based on large-scale corpora (Zhou and Xu, 2015; Niu et al., 2021; Tan et al., 2018). For example, the well-known pre-trained model Bidirectional Encoder Representations from Transformers (BERT) is based on a large-scale corpus (Devlin et al., 2018) and uses unsupervised learning of a masked language model to initiate a deep labeling method for pre-trained models, thus reducing dependence on well-designed task-specific architectures. BERT performs better than other models and has achieved the best results in 11 NLP test tasks (Huang et al., 2019). In a recent study, Song et al. (2020) introduced a novel method for recognizing local drug names in Xinjiang using the BERT-BiLSTM-CRF language model. The results showed that this approach effectively enhanced the performance of existing methods in practical applications. Additionally, Li W. et al. (2022) developed a BERT-BiLSTM-CRF model for accurately identifying named entities in the education section of public opinion data. Their model achieved impressive precision, recall, and F1 score values, improving performance in the named entity recognition task.

The extensive amount of TCM data also necessitates the deep application of IE technology. In recent years, ML and DL have been increasingly utilized in TCM research to automatically extract medical texts. Some scholars have applied general algorithms such as BERT base and Bi-LSTM to automatically extract ancient medical texts and electronic medical records (Hui et al., 2020; Chen et al., 2022; Liu et al., 2022). For example, Liu et al. (2022) have tested a pre-trained BERT model based on the traditional corpus of Sikuquanshu under the pre-training-fine-tuning paradigm. They have also constructed the software of named entity recognition based on SikuBERT, which verifies the feasibility of the model and provides a reference for further text mining and utilization of Chinese classics. To improve the effectiveness of electronic medical record named entity recognition, Chen et al. (2022) have proposed a hybrid neural network model based on MC-BERT. They extract the text’s local features and multilevel sequence interaction information to better represent electronic medical record text.

Preliminary research into the auto extraction of clinical evidence has shown promising results in the context of TCM (Hu et al., 2020). However, there are some limitations to previous studies. Scholars have often focused on abstracts and titles rather than the full texts, or have only studied one type of entity extraction, such as the number of participants or drug names, which is far from meeting the requirements of true automatic extraction of medical literature. Extraction models with both broad data coverage and excellent accuracy are still relatively lacking. Furthermore, the publicly available datasets and annotated corpora which are often used in general-purpose fields, are not always available in TCM research.

In this study, we aimed to develop a deep learning model that can automatically extract evidence-based information from TCM RCTs. This work would help us obtain evidence elements with greater accuracy and convenience in TCM. To extract complete information, including the title, abstract, and total document, we tried to construct a labelled literature dataset on TCM RCTs. This study aims to fill the gap of extracting only abstract and title information in TCM information extraction and provide a way to extract large-scale Chinese medicine literature automatically. Our ultimate goal is to expedite the process of structuring TCM information.

## Methods

### Overview

Here, we introduce the advanced pre-training language model Evi-BERT, which adopts both BERT and Bidirectional LSTM-CRF for evidence extraction in TCM RCTs. First, we construct a manually annotated dataset of TCM clinical literature, which contains 24,244 articles and abstracts. This dataset fills an important gap in the TCM field, as there is currently no dedicated research corpus. Next, we introduce Evi-BERT as a model to extract evidence information in TCM RCTs. This model consists of a BERT layer, an LSTM layer, and a CRF layer. To further improve the accuracy of evidence extraction, a regular expression (RE) method was combined with the Evi-BERT model.

### Electronic databases search

We conducted a systematic search in electronic databases, including the China National Knowledge Infrastructure (CNKI), WanFang Data, and VIP databases, covering the period from January 1, 1987 to December 30, 2020.

We searched Mandarin TCM clinical articles related to 11 target diseases, for which TCM is commonly used as an effective form of treatment. These 11 target diseases were selected from a list of diseases with exceptional TCM treatment effects recognized by the national administration of TCM. The 11 target diseases, including stroke, colorectal cancer, coronary heart disease, heart failure, chronic obstructive pulmonary disease, diabetes, diabetic nephropathy, osteoarthritis, obesity, rheumatoid arthritis, and diarrhea, are prevalent illnesses with extensive interest.

The search was performed by two authors (LH and LY) using keywords related to the target diseases and clinical studies. For example, we used the terms “type 2 diabetes” and “Chinese medicine” along with their related synonyms to search for articles on diabetes. The search formula used in CNKI was: SU = [糖尿病(diabetes) + 消渴(Xiaoke)] and SU = [中药(Chinese medicine) + 中成药(proprietary Chinese medicine) + 中草药(Chinese herbal medicine) + 草药(Herbal medicine) + 针灸(acupuncture) + 艾灸(moxibustion) + 中医疗法(Chinese medicine therapy)].

### Eligibility criteria

The titles and abstracts of all retrieved studies were screened independently by two expert authors (LH and LY) to identify relevant articles. Any disagreements during the screening process was resolved by the principal investigator of the study (HD). Articles were considered for inclusion if they met the following criteria: (1) published in Chinese with desired outcomes reported, (2) using at least one TCM therapy for the target disease, (3) reported as RCT-type studies. Studies were excluded if they were published in the form of a review, systemematic review, report, short communication, letter to editor, methodology, or editorial.

### RCT evidence selection

Following the principles of evidence-based medicine, RCT literature typically follows the five categories in the Population, Intervention, Comparison, Outcome, and Study design (PICOS) framework (Higgins et al., 2023). In this study, we selected ten elements from the five categories as extraction objects.

**Figure 1:**
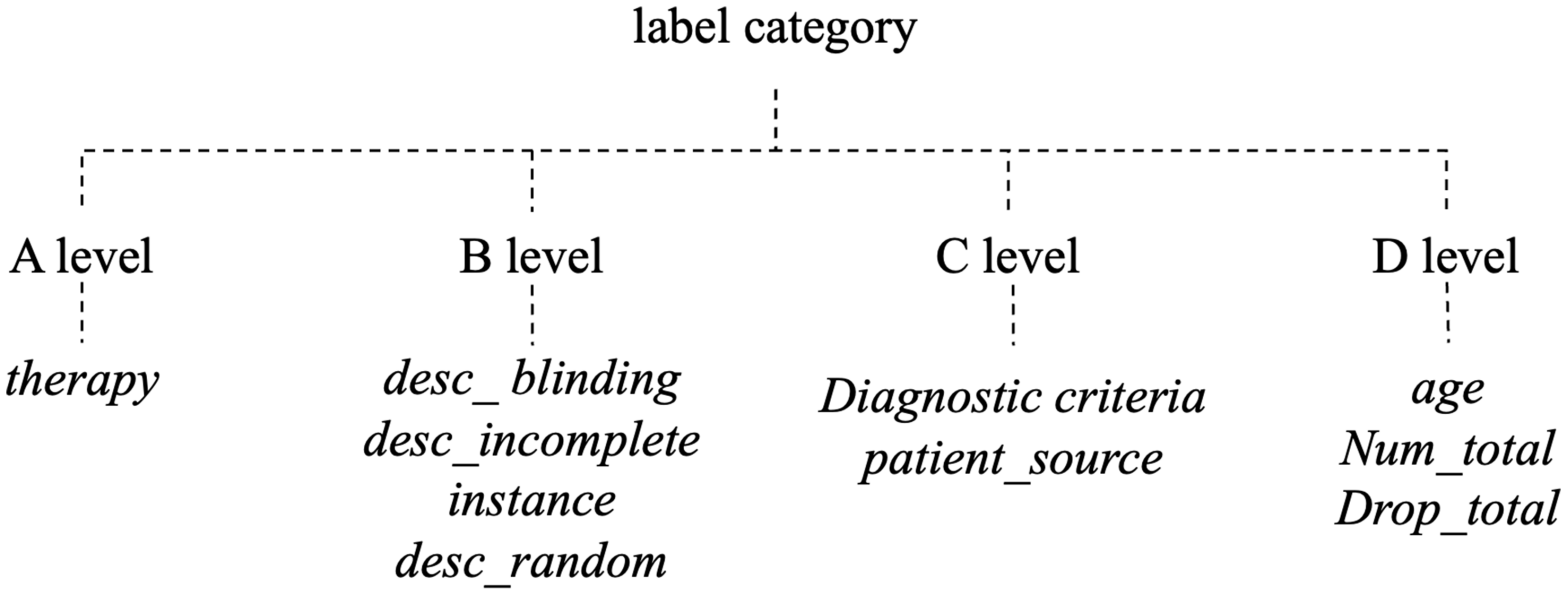
Evidence element output types. We represented the element output states through four different levels: A-levels were long sentences, B-levels were short sentences, C-level were phrases, and D-levels were numbers.

For the population category, we selected the total number and age distribution of participants, the source of participants, and the diagnostic criteria. For the intervention and comparison categories, we chose the therapy. For outcomes, we selected the occurrence of adverse effects and the shedding number. Finally, in the study design category, we chose missing data, blinded study design, and randomized study design. A detailed account of the relationship between each element and its corresponding category is provided in Supplementary Table S1. Depending on the output content, we categorized the ten evidence extraction elements into one of four types, consisting of one A-level class (representing sentence classes), four B-level classes (about short sentence classes), two C-level classes (associated with phrase classes), and three D-level classes (relating to number classes) (Figure 1).

### Building a domain corpus

There was no appropriate published corpus available for our research. Thus, we constructed labeling specifications for ten evidence elements tailored to our research needs based on TCM RCT characteristics. This process involved formulating annotation specifications and labeling entity names and content.

Primary labeling specifications are developed by analyzing the characteristics of the ten extracted elements. Annotators with medical backgrounds pre-labeled a batch of 200 articles in a randomized manner. By reviewing the annotation content and resolving discrepancies, experts and members of the standard-setting team finalized the labeling specifications.

To ensure the accuracy of our annotations, we designated an expert in a supervisory role, an expert well-versed in evidence-based medicine, two specialists with a background in clinical research, and included the input of 15 medical graduates and undergraduates. Before proper labeling, the person in charge provided a demonstration and explanation to annotators regarding the objects and standards requiring annotation. Two annotators marked the same batch of documents to ensure accuracy and uniformity, with any uncertain content highlighted for special attention. A third annotator performed a uniform check of the annotation results. In the case of disputes, the annotator notified the person in charge of the expert discussion to determine the final annotation content.

**Figure 2:**
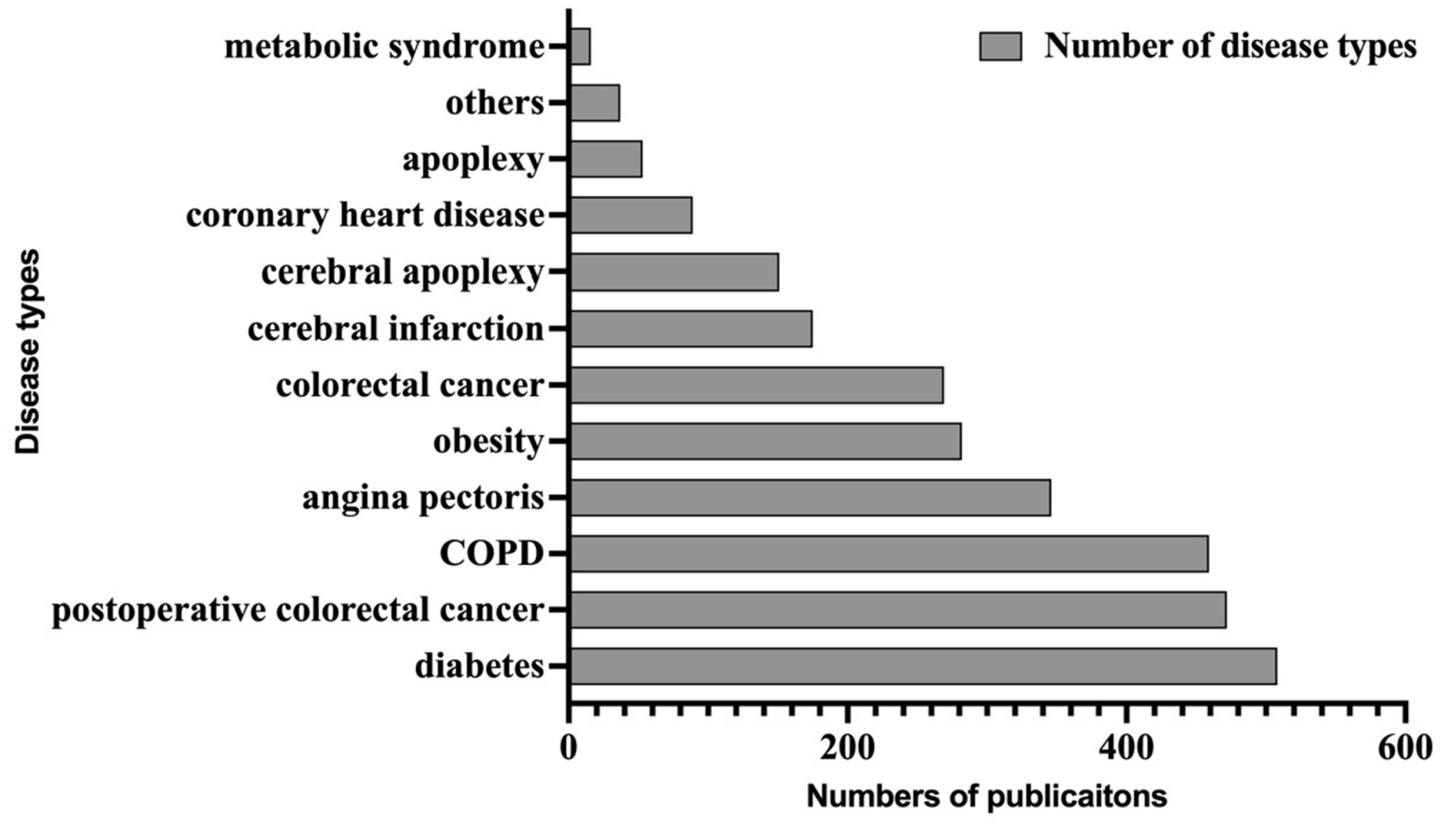
Number of disease types included in the corpus.

The labeling task involved 10,266 Chinese RCT paragraphs, resulting in a training set that included 24,244 pieces of data. We present the final labeling entity object information in Supplementary Table S2 and display some examples of the corpus in Supplementary Table S3. The attachment datas provide examples of entity type naming and tagging results that required tagging elements. The types of diseases involved in the corpus annotation were shown in Figure 2. We open-sourced our annotated publications dataset in the git-hub.^1^

#### Inter-rater reliability assessment

We employed two evaluators to assess the inter-rater reliability of the annotation process, using Cohen’s kappa score to evaluate consistency. Specifically, the annotation results were categorized into two classes: “accurate” and “ambiguous.” The evaluation design met the requirements for using Cohen’s kappa coefficient.

Due to the large volume of annotated data, we randomly selected 10% of the data for evaluation, resulting in a sample of 2,424 data points containing ten evidence elements. The results of the consistency evaluation are presented in the Table 1.

**Table 1:** Consistency check of the annotation process.

	B:accurate	B:ambiguous	B: total
A: accurate	**1746 (72.02%)** ^1^	118	1864
A: ambiguous	56	**504 (20.79%)** ^2^	560
A: total	1802	622	2,424

^1^ 72.02% (1,746/2,424) were deemed accurate by both evaluators.^2^ 20.79% (504/2,424) were considered ambiguous by both evaluators.

The results indicate that out of the 2,424 annotated data, 72.02% (1,746/2,424) were deemed accurate by both evaluators, while 20.79% (504/2,424) were considered ambiguous by both. A consistency test of the evaluators’ results yielded a Cohen’s kappa coefficient of 0.806, indicating a statistically significant agreement (*p* < 0.001) and a “very good” level of consistency. This finding demonstrates a high degree of agreement between Evaluator A and Evaluator B in their annotations, validating the reliability of the annotation process.

For machine learning training, we used a BIO for labeling, with B for the first word of an entity, I for the middle word of the entity, and O for anything that was not an entity. The markers were defined with a total of 31 tags, details can be found in Supplementary Datasheet S2.

We labeled raw text by cutting it into training samples with a maximum length of 128 characters per line to ensure that the trained corpus adhered to the maximum token length of the model. A separator line separated the labeled data to distinguish the general data corresponding to each article. Our approach can be applied to other domains of medicine and contribute to the advancement of NLP in medicine.

### Ethical considerations

This study involves a secondary information extraction of data previously published in academic journals. All data were collected anonymously.

### Model training and evaluation metrics

#### Model description

Our novel Evi-BERT model included a BERT layer, LSTM layer, and CRF layer. We used BERT for pre-training, and then input the trained word vectors into a LSTM for feature extraction. We then combined the output features of the neural network and finally corrected the prediction results using CRF. The model introduction were showed in Supplementary Datasheet S2. When fine-tuning the pre-training model, we added the manually labeled internal corpus.

#### Regulation extraction

At the end of this algorithm, we added a series of rule extraction formulas mainly composed of RE as text filtering and delicate extraction layers. Extracting rules is a reliable technique for extracting information from paragraphs and sentences. We obtained related statements containing extracted objects from source literature and then summarised the extraction rule set as PATTERN = {Pattern1, Pattern2, …}. The study focused on a total of 10 objects and summarized 113 rules, which were jointly summarized and completed by experts in TCM. The actual structure and schema examples of the rule sets are available in the Supplementary Datasheet S3.

**Figure 3:**
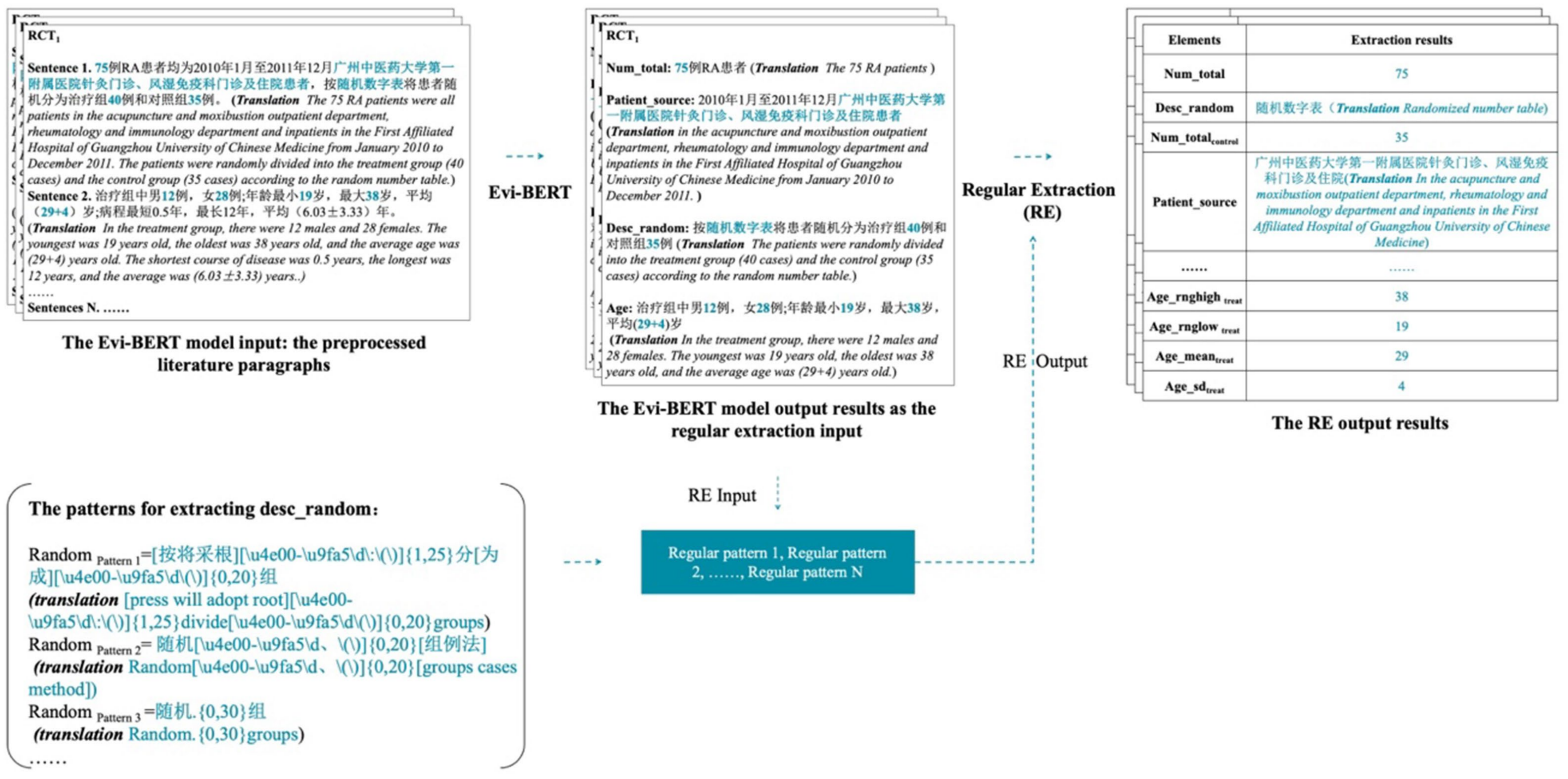
A demonstration of the input and output of the Evi-BERT model combined with rule extraction.

During the acquisition process, the output result of Evi-BERT serves as input for rule extraction. A regular expression is applied to match, judge, and split the output from the deep learning model. The final result is outputted with a finer level of granularity. The accompanying figure demonstrates this process with the des_random element’s input and output (Figure 3).

We used the sentence output from the pre-trained model as upstream inputs to the rule library and filtered them through the rule extraction formula to obtain finer-grained entity outputs. This process allows for errors in individual, unrelated characters. When the model is output, the result accuracy may be reduced due to character extraction redundancy. However, in the practical application of clinical evidence, if the redundant characters do not affect the overall information reading, the results can still be judged to be correct. For example, in the output “浙江中医学院附属新华医院杭州(Xinhua Hospital affiliated to Zhejiang College of Traditional Chinese Medicine hangzhou),” there is a redundancy of “杭州(hangzhou),” but it does not affect the understanding of this sentence. We determined that the sentence results were extracted accurately.

#### Experimental setting

We choose BERT-CRF (Hu et al., 2022) and Bi LSTM-CRF (Wang et al., 2023) as baseline models to compare with our novel Evi-BERT model. The model parameters adjusting showed in Supplementary Table S4. The training–testing dataset split ratio is 9:1. The optimizer used AdamW, and the lab environment and parameter settings used for model training were as follows:

*Processor (CPU): Intel Xeon-Gold 5,118 (2.3GHz/12-core/105 W);*

*Memory (RAM): 64G DDR4-2666 MT/s*

*System type: 64-bit operating system*

*Operating system: Linux*

*Programming language: Python 3.6*

*Integrated development environments: Visual Studio Code and Anaconda*

### Evaluation metrics

We used three of the most common metrics for sequence labeling tasks to evaluate the models’ robustness: Recall, Precision, and F1.

There were four model prediction results: TP, true positive; FP, false positive; TN, true negative; and FN, false negative (Table 2).

**Table 2:** Model prediction results.

		Real results	
		Positive	Negative
Predict results	Positive	TP	FP
	Negative	FN	TN

Recall is defined in Equation 1:


(1) $$\text{Recall}=\frac{TP}{\left(TP+FN\right)}$$

Precision is defined in Equation 2:


(2) $$\text{Precision}=\frac{TP}{\left(TP+FP\right)}$$

The F1 score is defined in Equation 3:


(3) $$F1\text{ score}=\frac{2*\text{Precision}*\text{Recall}}{\text{Precision}+\text{Recall}}$$

## Results

We searched and downloaded 48,523 TCM RCT clinical articles on these 11 target diseases from the electronic databases for information extraction. The number of documents of each disease type are provided in the Supplementary Table S5. All the original PDF files were transformed into machine-recognizable texts using a hybrid of machine vision and OCR techniques, including pyMuPDF^2^ and OpenCV.^3^ Each paper was named with the form “NUMBER_YEAR_AUTHOR_TITLE”.

We obtained the extraction results shown in Table 3. The addition of the BERT pre-training model significantly improved the effectiveness of a model named entity recognition. The precision, recall, and F1 values of Evi-BERT and BERT-CRF using the BERT pre-training model were considerably enhanced compared with Bi LSTM-CRF. These findings indicate that BERT has robust semantic representation abilities, which can greatly enhance the downstream recognition task performance. Compared with other named entity recognition methods, the Evi-BERT model proposed in this paper includes specific improvements—i.e., the addition of our newly generated internal corpus to fine-tune the model. Our Evi-BERT model has a Precision value of 0.643, an Recall value of 0.605, and an F1 value of 0.622 in entity recognition of the ten evidence elements, as using BERT as the pre-training word vector model fully allows our model to fully consider contextual semantic information when generating word vectors.

**Table 3:** Experimental evidence element extraction results.

Evaluation metrics		Model			
	RE only	BERT-CRF	Bi LSTM-CRF	Evi-BERT	Evi-BERT + RE
Precision	0.564	0.547	0.533	0.643	0.926
Recall	0.603	0.503	0.498	0.605	0.952
F1 score	0.572	0.524	0.515	**0.622** ^1^	**0.938** ^2^

^1^ The Evi-BERT model has an F1 value of 0.622 in entity recognition of the ten evidence elements.^2^ The Evi-BERT model combined RE has an F1 value of 0.938 in entity recognition of the ten evidence elements.

While its precision is commendable, its recall rate may be somewhat lacking. However, when combined with deep learning, this approach can significantly enhance accuracy and prevent misidentification.

Evi-BERT+RE can make up for the shortcomings of the BERT model in terms of extracting fine-grained information. Combining Evi-BERT with RE techniques improves precision, recall, and F1 compared with Evi-BERT alone because RE technology can extract language features in specific domain tasks more accurately without having to maintain a balance between accuracy and recall.

We also tested the results of document processing using rule extraction alone. The alone RE method has a Precision value of 0.564, a Recall value of 0.603, and an F1 value of 0.572. The reason for the low accuracy and recall value is that it is difficult to include all the statement paradigms using the rule set alone, and the labour cost is huge and inefficient. Combining Evi-BERT with RE techniques can more accurately locate the target element, The rule set only needs to match in sentences or phrases that are already anchored to the range, which greatly increases the accuracy of rule set matching. In practical application scenarios, entity recognition requires the output of accurate and unique standard results for downstream data analysis or calculations, and incorporating RE technology can significantly increase a model’s reliability. For example, we employed the Evi-BERT model to recognize sentences such as “treatment group age” and “control group age,” which were then used as input for fine-grained extraction of the “age” of men and women in each group using RE techniques.

## Discussion

### Principal findings

In this study, we introduce an Evi-BERT model which can accurately extract evidence elements from TCM-RCT literature. We manually labeled 24,244 database items to generate an internal corpus which fine-tuned the model. Compared with the baseline BiLSTM-CRF and BERT-CRF models, Evi-BERT showed superior precision, recall, and F1 scores. We found that the main types of errors in the output of deep learning models was noise introduced by imprecise terminology in the literature, leading to unclear boundaries for the entities being output. After incorporating real-world application scenarios, We next added RE techniques to Evi-BERT, applying the knowledge engineering method to refine the granularity of information extracted by the model. The Evi-BERT+RE showed significant improvement in results compared to the Evi-BERT-only model, with a Precision value of 0.926, an Rrecall value of 0.952, and an F1 value of 0.938. From the findings, it is clear that the RE method effectively addresses the accuracy issues observed in Evi-BERT’s output. Employing a pattern-matching-based RE method can aid deep learning models in capturing potentially overlooked information. In the practical application of clinical evidence extraction, researchers prioritize result accuracy over achieving a balance between precision and recall. The transparent operational strategy of the RE method ensures the reliability of the extraction outcomes, despite some sacrifice in recall rate.

### Comparison to prior work

Because of differences in journals’ designated formatting requirements and authors’ writing styles, RCT meta-analysis has traditionally relied on manual extraction, which has data breadth limitations and is less efficient. Furthermore, because of document recognition and difficulties in obtaining full literature reports, researchers usually focus on title and abstract extraction rather than full literature searches (Karystianis et al., 2023). Thus, full-text named entity recognition has remained a significant challenge in TCM meta-analysis research (Wang et al., 2020a). This study represents the first application of a pre-training linguistic model dominated by BERT for the structured extraction of literature from the full clinical text of TCM RCTs using a combination of optical character recognition technology and knowledge engineering methods.

In named entity recognition, in addition to considering the robustness of the model, the availability of output results is also one of the goals of model training. The extraction and analysis of medical evidence requires the identification of high-level (e.g., summaries and headings) and low-level (e.g., patients’ number and ages) data (Adnan and Akbar, 2019). Low-level data, as high-quality structured data, is critical for analyzing and understanding each paper’s content. Traditional knowledge engineering methods, such as rule and dictionary-based methods, are susceptible to limitations such as high coupling to the target domain and poor portability (Wang et al., 2020b). However, by predefining all synonyms and variants, the knowledge extraction method shows high accuracy when extracting high-quality structured data. However, its extreme robustness when pursuing training results may lead to misunderstandings of textual meaning and/or unique expressions, especially in TCM literature. Therefore, combining the RE method with deep learning may be the best choice to obtain accurate structured data in TCM research.

### Limitations

Despite the advantages of automatic extraction procedures, this study has several limitations:

Firstly, the quality of the manually labelled corpus used for model training may have contributed to the low F1 score. The lack of a uniform specification or standard for literature annotation tasks increases the likelihood of errors between annotation corpora, highlighting the need for further research on annotation specifications for different types of elements to reduce errors and improve machine learning training performance. It is essential to elevate the standard of homogeneity and expand the corpus of annotated data.

Secondly, while the selection of literature on eleven diseases was based on the number of literature in the catalogue of TCM internal medicine and the scientific and technological database, there was no further analysis of the literature content or optimization of inclusion criteria. As a result, the selection of literature may have been relatively broad and biased. Additionally, the rule base constructed is limited to the 11 diseases studied, and further research is needed to extend it to more diseases.

Thirdly, due to the need for substantial amounts of manpower and time, only a portion of the literature can be sampled to verify Evi-BERT+RE results. The addition of more verification data may improve the reliability of results in future studies. While the Evi-BERT+RE model demonstrates remarkable improvements in precision, recall, and F1 scores, the significant leap in performance metrics may raise questions about potential overfitting. Further efforts are warranted to enhance the robustness and generalizability of the study. This could involve the development of an automatic program with a verification function and the exploration of the practical application of large language models in similar tasks.

Finally, the literature evidence for TCM RCT also includes a wide range of important information that is difficult to auto-extract, such as the name of the prescription, drug composition, drug use frequency, and changes in outcome indicators. Consequently, there is a pressing need to develop a more comprehensive in-depth extraction model that can improve the comprehension of TCM RCTs.

### Future directions

While machine and deep learning have revolutionized many areas of NLP, extraction information in TCM is still in the first stages. We note that as of 2021, electronic databases include more than 3.9 million pieces of literature on TCM, with the majority in the Chinese language. As such, our study can aid the structured extraction of Chinese literature and the modernization of TCM approaches. To enhance the model’s generalizability to unseen data, users can be guided to set the initial document format to meet specific requirements. Additionally, output data validation procedures can be incorporated for intermediate results. In the next step, we hope to include more labeled data with low heterogeneity, and add an external knowledge base of Chinese herbal medicine, acupuncture points, and Chinese herbal prescription collections to enhance the capacity of the model.

Additionally, we hope the structured clinical evidence and structured knowledge extracted from this study will help other researchers build large language models in TCM. Important future directions include: generating unified specifications for annotation tasks in TCM RCT, extending the extraction elements in detail, and improving the accuracy of information extraction.

## Data availability statement

The datasets presented in this study can be found in online repositories. The names of the repository/repositories and accession number(s) can be found in the article/Supplementary material.

## Author contributions

YLi: Writing – original draft, Writing – review & editing. ZL: Funding acquisition, Methodology, Project administration, Writing – original draft. YLiu: Supervision, Validation, Writing – original draft. HL: Validation, Writing – review & editing. JQ: Data curation, Methodology, Writing – review & editing. DH: Project administration, Writing – review & editing.

## Footnotes

^1^ https://github.com/yizhen-buaa/Annotated-dataset-of-TCM-clinical-literature

^2^ https://pymupdf.io

^3^ https://opencv.org

## References

[ref1] Adnan K. Akbar R. (2019). Limitations of information extraction methods and techniques for heterogeneous unstructured big data. Int. J. Eng. Business Manag. 11:184797901989077. doi: 10.1177/1847979019890771

[ref2] Chen Y. P. Lo Y. H. Lai F. Huang C. H. (2021). Disease concept-embedding based on the self-supervised method for medical information extraction from electronic health records and disease retrieval: algorithm development and validation study. J. Med. Internet Res. 23:e25113. doi: 10.2196/25113, PMID: 33502324 PMC7875703

[ref3] Chen P. Zhang M. Yu X. Li S. (2022). Named entity recognition of Chinese electronic medical records based on a hybrid neural network and medical MC-BERT. BMC Med. Inform. Decis. Mak. 22, 1–13. doi: 10.1186/s12911-021-01695-4, PMID: 36457119 PMC9714133

[ref4] Devlin J. Chang M. W. Lee K. Toutanova K. (2018). Bert: pre-training of deep bidirectional transformers for language understanding. arXiv. doi: 10.48550/arXiv.1810.04805

[ref5] Foufi V. Timakum T. Gaudet-Blavignac C. Lovis C. Song M. (2019). Mining of textual health information from Reddit: analysis of chronic diseases with extracted entities and their relations. J. Med. Internet Res. 21:e12876. doi: 10.2196/12876, PMID: 31199327 PMC6595941

[ref6] Graves A. Schmidhuber J. (2005). Framewise phoneme classification with bidirectional LSTM and other neural network architectures. Neural Netw. 18, 602–610. doi: 10.1016/j.neunet.2005.06.042 16112549

[ref7] Guo E. Gupta M. Deng J. Park Y. J. Paget M. Naugler C. (2024). Automated paper screening for clinical reviews using large language models: data analysis study. J. Med. Internet Res. 26:e48996. doi: 10.2196/48996, PMID: 38214966 PMC10818236

[ref8] Higgins J Thomas J Chandler J Cumpston M Li T Page M Cochrane Training: The Cochrane Collaboration (2023). Availabel at: Cochrane Handbook for Systematic Reviews of Interventions URL: https://training.cochrane.org/handbook/current (Accessed January 19, 2024).

[ref9] Hochreiter S. Schmidhuber J. (1997). Long short-term memory. Neural Comput. 9, 1735–1780. doi: 10.1162/neco.1997.9.8.1735 9377276

[ref10] Hu F. Li L. Huang X. Yan X. Huang P. (2020). Symptom distribution regularity of insomnia: network and spectral clustering analysis. JMIR Med. Inform. 8:e16749. doi: 10.2196/16749 32297869 PMC7193440

[ref11] Hu S. Zhang H. Hu X. Du J. (2022). “Chinese Named Entity Recognition based on BERT-CRF Model” in 2022 IEEE/ACIS 22nd international conference on computer and information science (ICIS) (Zhuhai, China: IEEE), 105–108.

[ref12] Huang W Cheng X Wang T Chu W. Bert-based multi-head selection for joint entity-relation extraction. Natural Language Processing and Chinese Computing: 8th CCF International Conference; (2019); Dunhuang, China. Springer, Cham.

[ref13] Hui Y Du L Lin S Qu Y Cao D. Extraction and classification of TCM medical records based on BERT and bi-LSTM with attention mechanism. (2020) IEEE International Conference on Bioinformatics and Biomedicine (BIBM): 1626–1631; Seoul, Korea (South). IEEE.

[ref14] Kang T. Sun Y. Kim J. H. Ta C. Perotte A. Schiffer K.. (2023). Evidence map: a three-level knowledge representation for medical evidence computation and comprehension. J. Am. Med. Inform. Assoc. 30, 1022–1031. doi: 10.1093/jamia/ocad036, PMID: 36921288 PMC10198523

[ref15] Karystianis G. Simpson P. Lukmanjaya W. Ginnivan N. Nenadic G. Buchan I.. (2023). Automatic extraction of research themes in epidemiological criminology from PubMed abstracts from 1946 to 2020: text mining study. JMIR Form Res. 7:e49721. doi: 10.2196/49721, PMID: 37738080 PMC10559193

[ref16] Kim G. Lee C. Jo J. Lim H. (2020). Automatic extraction of named entities of cyber threats using a deep bi-LSTM-CRF network. Int. J. Mach. Learn. Cybern. 11, 2341–2355. doi: 10.1007/s13042-020-01122-6

[ref17] Li W. Du Y. Li X. Chen X. Xie C. Li H.. (2022). UD_BBC: named entity recognition in social network combined BERT-BiLSTM-CRF with active learning. Eng. Appl. Artif. Intell. 116:105460. doi: 10.1016/j.engappai.2022.105460

[ref18] Li Y. Hui L. Zou L. Li H. Xu L. Wang X.. (2022). Relation extraction in biomedical texts based on multi-head attention model with syntactic dependency feature: modeling study. JMIR Med. Inform. 10:e41136. doi: 10.2196/41136, PMID: 36264604 PMC9634522

[ref19] Li J Wang T Zhang W. An improved Chinese named entity recognition method with TB-LSTM-CRF. (2020) 2nd Symposium on Signal Processing Systems. 2020: 96-100; Guangdong, China.

[ref21] Liu K. El-Gohary N. (2017). Ontology-based semi-supervised conditional random fields for automated information extraction from bridge inspection reports. Autom. Constr. 81, 313–327. doi: 10.1016/j.autcon.2017.02.003

[ref22] Liu J. Feng Y. Wang D. Hu H. Zhang Y. (2022). Research on SikuBERT-enhanced entity recognition of historical records from the perspective of digital humanity. Library Tribune 42, 61–72.

[ref23] Liu Z. Liu Y. Xu H. He L. Chen Y. Fu L.. (2017). Effect of electroacupuncture on urinary leakage among women with stress urinary incontinence: a randomized clinical trial. JAMA 317, 2493–2501. doi: 10.1001/jama.2017.7220, PMID: 28655016 PMC5815072

[ref24] Liu Z. Yan S. Wu J. He L. Li N. Dong G.. (2016). Acupuncture for chronic severe functional constipation: a randomized trial. Ann. Intern. Med. 165, 761–769. doi: 10.7326/M15-3118 27618593

[ref25] Malec S. A. Taneja S. B. Albert S. M. Shaaban C. E. Karim H. T. Levine A. S.. (2023). Causal feature selection using a knowledge graph combining structured knowledge from the biomedical literature and ontologies: a use case studying depression as a risk factor for Alzheimer’s disease. J. Biomed. Inform. 142:104368. doi: 10.1016/j.jbi.2023.104368, PMID: 37086959 PMC10355339

[ref26] Maruf S. Saleh F. Haffari G. (2021). A survey on document-level neural machine translation: methods and evaluation. ACM Comput. Surv. 54, 1–36. doi: 10.1145/3441691

[ref27] Niu Z. Zhong G. Yu H. (2021). A review on the attention mechanism of deep learning. Neurocomputing 452, 48–62. doi: 10.1016/j.neucom.2021.03.091

[ref28] Song Y. Tian S. Yu L. (2020). A method for identifying local drug names in Xinjiang based on BERT-BiLSTM-CRF. Aut. Control Comp. Sci. 54, 179–190. doi: 10.3103/S0146411620030098

[ref29] Stylianou N. Razis G. Goulis D. G. Vlahavas I. (2020). EBM+: advancing evidence-based medicine via two level automatic identification of populations, interventions, outcomes in medical literature. Artif. Intell. Med. 108:101949. doi: 10.1016/j.artmed.2020.101949, PMID: 32972669

[ref30] Sugimoto K. Wada S. Konishi S. Okada K. Manabe S. Matsumura Y.. (2023). Extracting clinical information from Japanese radiology reports using a 2-stage deep learning approach: algorithm development and validation. JMIR Med. Inform. 11:e49041. doi: 10.2196/49041 37991979 PMC10686535

[ref31] Tan C. Sun F. Kong T. Zhang W. Yang C. Liu C. (2018). “A survey on deep transfer learning” in Artificial neural networks and machine learning–ICANN 2018 sep 27. Lecture notes in computer science(). eds. Kůrková V. Manolopoulos Y. Hammer B. Iliadis L. Maglogiannis I., vol. 11141 (Cham: Springer).

[ref32] Tu J. F. Yang J. W. Shi G. X. Yu Z. S. Li J. L. Lin L. L.. (2021). Efficacy of intensive acupuncture versus sham acupuncture in knee osteoarthritis: a randomized controlled trial. Arthritis Rheumatol. 73, 448–458. doi: 10.1002/art.41584, PMID: 33174383

[ref20] Wang J. Lin C. Li M. Zaniolo C. (2020a). Boosting approximate dictionary-based entity extraction with synonyms. Inf. Sci. 530, 1–21. doi: 10.1016/j.ins.2020.04.025

[ref33] Wang J. Deng H. Liu B. Hu A. Liang J. Fan L.. (2020b). Systematic evaluation of research progress on natural language processing in medicine over the past 20 years: bibliometric study on PubMed. J. Med. Internet Res. 22:e16816. PMID: 32012074. doi: 10.2196/16816, PMID: 32012074 PMC7005695

[ref34] Wang W. Li X. Ren H. Gao D. Fang A. (2023). Chinese clinical named entity recognition from electronic medical records based on multisemantic features by using robustly optimized bidirectional encoder representation from transformers Pretraining approach whole word masking and convolutional neural networks: model development and validation. JMIR Med. Inform. 11:e44597. doi: 10.2196/44597 37163343 PMC10209791

[ref35] Xu H. Hu B. (2022). Legal text recognition using LSTM-CRF deep learning model. Comput. Intell. Neurosci. 2022, 1–10. doi: 10.1155/2022/9933929 PMC8947905 35341203

[ref36] Zhang T. Huang Z. Wang Y. Wen C. Peng Y. Ye Y. (2022). Information extraction from the text data on traditional Chinese medicine: a review on tasks, challenges, and methods from 2010 to 2021. Evid. Based Complement. Alternat. Med. 2022, 1–19. doi: 10.1155/2022/1679589 PMC9122692 35600940

[ref37] Zhou J Xu W. End-to-end learning of semantic role labeling using recurrent neural networks. Proceedings of the 53rd Annual Meeting of the Association for Computational Linguistics and the 7th International Joint Conference on Natural Language Processing (1: Long Papers). (2015): 1127–1137.

[ref38] Zirikly A. Desmet B. Newman-Griffis D. Marfeo E. E. McDonough C. Goldman H.. (2022). Information extraction framework for disability determination using a mental functioning use-case. JMIR Med. Inform. 10:e32245. doi: 10.2196/32245 35302510 PMC8976250

