# Supplementary material for: Automated information extraction model enhancing traditional Chinese medicine RCT evidence extraction (Evi-BERT): algorithm development and validation
Source: Front Artif Intell. 2024 Aug 15;7:1454945. doi: 10.3389/frai.2024.1454945 (PMC11358118; doi:10.3389/frai.2024.1454945)
Supplement: Supplementary file 1 [file Data_Sheet_1.docx]

**Multimedia Appendix 1**

**Attachment files**

**Attachment Table 1. Relationship between elements and corresponding categories**

| **We included ten elements from five PICOS categories. Here, we list the abbreviations for each element in the table and explain their meaning.** |
| --- |
| 1. Participants (P)   - Total number (Num_total)   *Total number of clinical study participants*   - Diagnostic criteria   Tcm_diagref  *Sources and entries of diagnostic criteria containing references to the diseases studied, and sources and entries of TCM symptom criteria (TCM section)*  Wm_diagref  *Sources and entries of diagnostic criteria containing references to the diseases studied (western medicine section)*   - Source of participants (Patient_source)   *Place of residence of clinical study subjects*   - Age distribution of participants (Age)   including: age_rnghigh, age_rnglow, age_mean and age_sd)  *Maximum, minimum, average, and standard deviation age of participants*  *Minimum age of participants*  2. Intervention (I)   - Therapy of intervention   *Contains treatment regimens for all groups*  3. Comparison(C)   - Therapy of comparison   *Contains treatment regimens for all groups*  4. Outcome(O)   - Occurrence of adverse effects (Instances)   *Reports of adverse reactions that occurred during clinical trials*   - Shedding number (Drop_total)   *Total number of subject dropouts during clinical trials*  5. Study-design(S)   - Missing data (Desc_incomplete)   *Missing data description*   - Blinded (Desc_blinding)   *Clinical trial design was blinded*   - Randomized (Desc_random)   *Clinical trial design was randomized* |

**Attachment Table 2. Number of manually pre-labeled entities for each evidence element**

| Series | Evidence element | Count |
| --- | --- | --- |
| 1 | Patient_source (PSrc) | 3,331 |
| 2 | Num_total (PNum) | 3,875 |
| 3 | Age (PAge/PAgeT/PAgeC) | 5,267 |
| 4 | Desc_random (PRnd) | 3,116 |
| 5 | Desc_blinding (PBld) | 47 |
| 6 | Wm_diagref (StdW) | 1,910 |
| 7 | Tcm_diagref (StdC) | 1,191 |
| 8 | Therapy (TeyC/TeyT) | 4,590 |
| 9 | Instance (InsC/InsT) | 769 |
| 10 | Drop_total (NumDC/ NumDT) | 148 |
|  | total | 24,244 |

**Attachment Table 3. Examples of corpus annotation**

| **No.** | **Chinese RCT paragraphs** | **Elements** |
| --- | --- | --- |
| 1 | Clinical data 1. General data A total of 640 (**PNum**) patients were included in this study, all of whom were T2DM patients in the acupuncture and moxibustion Clinic of Nanjing University of TCM (**PSrc**) from January 2006 to December 2011. | 640 (**PNum**)  acupuncture and moxibustion Clinic of Nanjing University of TCM (**PSrc**) |
| 2 | Grouping and medication method: Experimental group, Liuwei Dihuang Soft Capsules, oral administration, 3 capsules per time, 2 times daily, Ginkgo biloba leaves, oral administration, 1 tablet per time, 3 times daily (**TeyT**). The control group received a placebo of Liuwei Dihuang Soft Capsule, orally taking 3 capsules twice a day, taking 1 tablet twice a day (T**eyC**). | Experimental group, Liuwei Dihuang Soft Capsules……3 times daily. (**TeyT**)；  The control group ……twice a day. (**TeyC**) |
| 3 | Observation of therapeutic effects 3.1. Criteria for efficacy evaluation Literature, the total clinical efficacy is determined based on clinical symptoms, signs, and symptom score improvement. 3.2 Results: In the control group, there were 3 cases of dropout due to self-selection and cooperation with other treatments during treatment (**NumDC**), while in the treatment group, there was 1 case of dropout due to not adhering to treatment during a business trip (**NumDT**). | there were 3 cases ……treatments during treatment (**NumDC**);  there was 1 case ……a business trip (**NumDT**) |
| 4 | Results 2.1 Comparison of clinical efficacy (see attached table) 2.2 Side effects: Two cases in the observation group experienced dizziness, but the symptoms were mild and did not affect treatment. No damage to liver, kidney, and hematopoietic system function was found (**InsT**). No significant adverse reactions were found in the control group (**InsC**). | Two cases in the observation group ……was found (**InsT**);  No significant …… the control group (**InsC**). |

**Attachment Table 4**. **Experimental parameter settings**

| **Parameter setting** | **Model** | | |
| --- | --- | --- | --- |
|  | ***BERT-CRF*** | ***Bi LSTM-CRF*** | ***Evi-BERT*** |
| Batch_size | 4 | 64 | 32 |
| Training learning rate | 3e-5 | 0.001 | 3e-5 |
| Epoch_num | 20 | 20 | 20 |

**Attachment Table 5. Scientific and technological bibliography and total database literature**

| **num** | **Literature disease type** | **Number of documents** |
| --- | --- | --- |
| 1 | diabetes | 2,478 |
| 2 | diabetic nephropathy | 4,072 |
| 3 | corpulent | 1,840 |
| 4 | chronic obstructive pulmonary disease (COPD) | 4,290 |
| 5 | knee osteoarthritis | 6,240 |
| 6 | rheumatoid arthritis | 2,608 |
| 7 | colorectal cancer | 1,094 |
| 8 | coronary heart disease | 10,660 |
| 9 | stroke | 69,92 |
| 10 | heart failure | 6,531 |
| 11 | pediatric diarrhea | 1,718 |
| **total** | **-** | **48,523** |
