# Supplementary material for: Automated information extraction model enhancing traditional Chinese medicine RCT evidence extraction (Evi-BERT): algorithm development and validation
Source: Front Artif Intell. 2024 Aug 15;7:1454945. doi: 10.3389/frai.2024.1454945 (PMC11358118; doi:10.3389/frai.2024.1454945)
Supplement: Supplementary file 2 [file Data_Sheet_2.docx]

**Multimedia Appendix 2**

Our novel Evi-BERT model included a BERT layer, LSTM layer, and CRF layer. We used BERT for pre-training, and then input the trained word vectors into a LSTM for feature extraction. We then combined the output features of the neural network and finally corrected the prediction results using CRF, as:

**
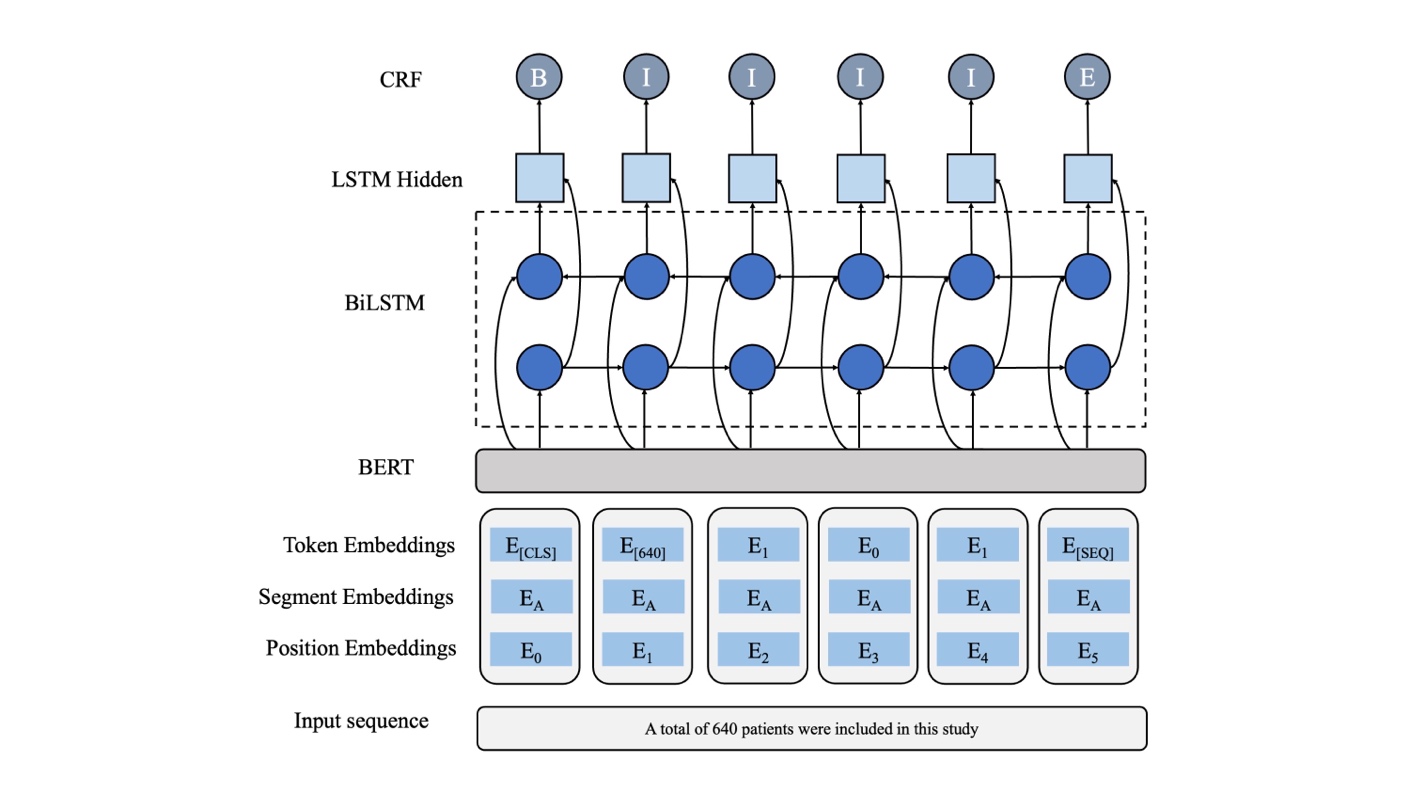
**

**Figure 1. Evi-BERT Model Structure**

The markers were defined with a total of 31 tags, details as:

O; B-PSrc，B-PNum，B-PAgeT，B-PAgeC，B-PAge，B-PRnd，B-PBld，B-StdW，B-StdC, B-TeyC, B-TeyT, B-InsT, B-InsC, B-NumDT, B-NumDC; I-PSrc，I-PNum，I-PAgeT，I-PAgeC，I-PAge，I-PRnd，I-PBld，I-StdW，I-StdC，I-TeyC, I-TeyT, I-InsT, I-InsC, I-NumDT, I-NumDC.
