# Supplementary material for: Automated information extraction model enhancing traditional Chinese medicine RCT evidence extraction (Evi-BERT): algorithm development and validation
Source: Front Artif Intell. 2024 Aug 15;7:1454945. doi: 10.3389/frai.2024.1454945 (PMC11358118; doi:10.3389/frai.2024.1454945)
Supplement: Supplementary file 3 [file Data_Sheet_3.docx]

### Multimedia Appendix 3

### In this attachment files, we listed the examples of pattern rules and running codes of five elements in our model. The five elements are: Desc_random, Desc_incomplete, Desc_blind, Age and Num_total

### 1. Desc_random pattern rules and codes

def getPatientRandom_middle(text):

    random_middle1 = re.search('[按将采根][\u4e00-\u9fa5\d\:\(\)]{1,25}分[为成][\u4e00-\u9fa5\d\(\)]{0,20}组',text)

    random_middle2 = re.search('[按将采根][\u4e00-\u9fa5\d\:\(\)]{1,25}分[为成][\u4e00-\u9fa5\d\(\)]{0,20}[组例]',text)

    random_middle3 = re.search('[按将采根][\u4e00-\u9fa5\d\:\(\)]{0,25}随机[\u4e00-\u9fa5\d\(\)]{0,20}[组例法]',text)

    random_middle4 = re.search('随机[\u4e00-\u9fa5\d、\(\)]{0,20}[组例法]',text)

    random_middle5 = re.search('随机.{0,30}组',text)

    random_middle6 = re.search('.{0,30}随机.{0,30}',text)

    random_middle = ''

    if random_middle1 != None:

        random_middle = random_middle1.group()

    elif random_middle2 != None:

        random_middle = random_middle2.group()

    elif random_middle3 != None:

        random_middle = random_middle3.group()

    elif random_middle4 != None:

        random_middle = random_middle4.group()

    elif random_middle5 != None:

        random_middle = random_middle5.group()

        #print (random_middle)

    elif random_middle6 != None:

        random_middle = random_middle6.group()

    else:

        random_middle = '未提及'

    return random_middle

### 2. Desc_incomplete pattern rules and codes

def getdrop_exp_amout(text):

    drop_exp_amout = ''

    drop_exp_amout1 = re.search('治疗组脱[失落]\d{1,3}例|治疗组\d{1,3}例脱落',text)

    drop_exp_amout2 = re.search('治疗组.+?脱[失落].+?\d{1,3}例|治疗组.+?\d{1,3}例脱落',text)

    if drop_exp_amout1 != None:

        drop_exp_amout = drop_exp_amout1.group()

    elif drop_exp_amout2 != None:

        drop_exp_amout = drop_exp_amout2.group()

    else:

        drop_exp_amout = '未提及'

    return drop_exp_amout

def getdrop_exp_amout_t(text):

    drop_exp_amout_t = ''

    drop_exp_amout1 = re.search('治疗组脱落(\d{1,3}例)',text)

    drop_exp_amout2 = re.search('治疗组(\d{1,3}例)脱落',text)

    if drop_exp_amout1 != None:

        drop_exp_amout_t = drop_exp_amout1.group(1)

    elif drop_exp_amout2 != None:

        drop_exp_amout_t = drop_exp_amout2.group(1)

    else:

        drop_exp_amout_t = '！人工识别'

    return drop_exp_amout_t

def getdrop_con_amout(text):

    drop_con_amout = ''

    drop_con_amout1 = re.search('对照组脱[失落]\d{1,3}例|对照组\d{1,3}例脱落',text)

    drop_con_amout2 = re.search('对照组.+?脱[失落].+?\d{1,3}例|对照组.+?\d{1,3}例脱落',text)

    if drop_con_amout1 != None:

        drop_con_amout = drop_con_amout1.group()

    elif drop_con_amout2 != None:

        drop_con_amout = drop_con_amout2.group()

    else:

        drop_con_amout = '未提及'

    return drop_con_amout

def getdrop_con_amout_t(text):

    drop_con_amout_t = ''

    drop_con_amout1 = re.search('对照组脱落(\d{1,3}例)',text)

    drop_con_amout2 = re.search('对照组(\d{1,3}例)脱落',text)

    if drop_con_amout1 != None:

        drop_con_amout_t = drop_con_amout1.group(1)

    elif drop_con_amout2 != None:

        drop_con_amout_t = drop_con_amout2.group(1)

    else:

        drop_con_amout_t = '！人工识别'

    return drop_con_amout_t

def get_TuoLuo(text):

    TuoLuo = ''

    example1 =  re.search('[未无].+?脱落',text)

    example3 =  re.search('\d{1,3}患者脱落',text)

    example4 = re.search('脱落\d{1,3}例',text)

    example5 = re.search('脱[失落]病例.+?。\n|脱落情况.+?。\n',text)

    if example1 != None:

        TuoLuo = example1.group()

    elif example3 != None:

        TuoLuo = example3.group()

    elif example4 != None:

        TuoLuo = example4.group()

    elif example5 != None:

        TuoLuo = example5.group()

    else:

        TuoLuo = '未提及'

    return TuoLuo

def getdesc_rob_incompltet(sum_tuoluo,drop_exp_amout,drop_con_amout):

    rob_incomplete = desc_rob_incomplete= ''

    if sum_tuoluo == '0例' or sum_tuoluo == '未脱落':

        rob_incomplete='Low'

        desc_rob_incomplete='无脱落数据'

    elif sum_tuoluo == '未提及':

        rob_incomplete = 'unclear'

    else:

        rob_incomplete= ''

        desc_rob_incomplete = drop_exp_amout + '；' + drop_con_amout

return rob_incomplete,desc_rob_incomplete

**3. Desc_blinding pattern rules and codes**

def getPatientBlind_m(text):

blind_m = ''

if re.search('.{0,10}[单双]盲.{0,10}',text) != None :

blind_m = re.search('.{0,10}[单双]盲.{0,10}',text).group()

return blind_m

#step2

def getPatientBlind_f(text):

blind = ''

if re.search('单盲', text) != None:

blind = '单盲'

elif re.search('双盲', text) != None:

blind = '双盲'

elif re.search('单盲|双盲', text) != None:

blind = re.search('单盲|双盲', text).group()

else:

blind = '未提及'

return blind

### 4. Num_total pattern rules and codes

def getgroupsexnumbers1(text):

    sexnumbers1 = re.search('治疗组.{0,5}?[男女]性{0,1}患{0,1}[儿者]{0,1}(\d{1,3})[例名位个人]{0,1}.{0,10}?[男女]性{0,1}患{0,1}[儿者]{0,1}(\d{1,3})[例名位个人]{0,1}',text)

    treamentGroupsexnumber1 = ''

    if sexnumbers1 != None:

        treamentGroupsexnumber1 = sexnumbers1.group(1)

    return treamentGroupsexnumber1

def getgroupsexnumbers2(text):

    sexnumbers1 = re.search('治疗组.{0,5}?[男女]性{0,1}患{0,1}[儿者]{0,1}(\d{1,3})[例名位个人]{0,1}.{0,10}?[男女]性{0,1}患{0,1}[儿者]{0,1}(\d{1,3})[例名位个人]{0,1}',text)

    treamentGroupsexnumber2 = ''

    if sexnumbers1 != None:

        treamentGroupsexnumber2 = sexnumbers1.group(2)

    return treamentGroupsexnumber2

def getgroupsexnumbers3(text):

    sexnumbers1 = re.search('对照组.{0,5}?[男女]性{0,1}患{0,1}[儿者]{0,1}(\d{1,3})[例名位个人]{0,1}.{0,10}?[男女]性{0,1}患{0,1}[儿者]{0,1}(\d{1,3})[例名位个人]{0,1}',text)

    contrastGroupsexnumber1 = ''

    if sexnumbers1 != None:

        contrastGroupsexnumber1 = sexnumbers1.group(1)

    return contrastGroupsexnumber1

def getgroupsexnumbers4(text):

    sexnumbers1 = re.search('对照组.{0,5}?[男女]性{0,1}患{0,1}[儿者]{0,1}(\d{1,3})[例名位个人]{0,1}.{0,10}?[男女]性{0,1}患{0,1}[儿者]{0,1}(\d{1,3})[例名位个人]{0,1}',text)

    contrastGroupsexnumber2 = ''

    if sexnumbers1 != None:

        contrastGroupsexnumber2 = sexnumbers1.group(2)

    return contrastGroupsexnumber2

def getothergroupsexnumbers1(text):

    sexnumbers2 = re.search('[\u4e00-\u9fa5]{2,5}组.{0,50}?[男女]性{0,1}患{0,1}[儿者]{0,1}(\d{1,3})[例名位个人]{0,1}.{0,10}?[男女]性{0,1}患{0,1}[儿者]{0,1}(\d{1,3})[例名位个人]{0,1}',text)

    otherGroupsexnumber1 = ''

    if sexnumbers2 != None:

        otherGroupsexnumber1 = sexnumbers2.group(1)

    return otherGroupsexnumber1

def getothergroupsexnumbers2(text):

    sexnumbers2 = re.search('[\u4e00-\u9fa5]{2,5}组.{0,50}?[男女]性{0,1}患{0,1}[儿者]{0,1}(\d{1,3})[例名位个人]{0,1}.{0,10}?[男女]性{0,1}患{0,1}[儿者]{0,1}(\d{1,3})[例名位个人]{0,1}',text)

    otherGroupsexnumber2 = ''

    if sexnumbers2 != None:

        otherGroupsexnumber2 = sexnumbers2.group(2)

    return otherGroupsexnumber2

def getnogroupsexnumbers1(text):

    nosexnumbers1 = re.search('其中[男女]性{0,1}患{0,1}[儿者]{0,1}(\d{1,3})[例名位个人]{0,1}.{0,10}[男女]性{0,1}患{0,1}[儿者]{0,1}(\d{1,3})[例名位个人]{0,1}',text)

    nosexnumber1 = ''

    if nosexnumbers1 != None:

        nosexnumber1 = nosexnumbers1.group(1)

    return nosexnumber1

def getnogroupsexnumbers2(text):

    nosexnumbers2 = re.search('其中[男女]性{0,1}患{0,1}[儿者]{0,1}(\d{1,3})[例名位个人]{0,1}.{0,10}[男女]性{0,1}患{0,1}[儿者]{0,1}(\d{1,3})[例名位个人]{0,1}',text)

    nosexnumber2 = ''

    if nosexnumbers2 != None:

        nosexnumber2 = nosexnumbers2.group(2)

    return nosexnumber2

def gettregroupnumbers(text):

    tregroupnumbers = re.search('治疗组.{0,20}?(\d{1,3})[例名位个人]',text)

    treamentGroupnumber = ''

    if tregroupnumbers != None:

        treamentGroupnumber = tregroupnumbers.group(1)

    return treamentGroupnumber

def getcongroupnumbers(text):

    congroupnumbers = re.search('对照组.{0,20}?(\d{1,3})[例名位个人]',text)

    contrastGroupnumber = ''

    if congroupnumbers != None:

        contrastGroupnumber = congroupnumbers.group(1)

    return contrastGroupnumber

def getgroupnumbers(text):

    groupnumbers1 = re.search('每组(\d{1,3})[例名位个人]',text)

    groupnumbers2 = re.search('每组各(\d{1,3})[例名位个人]',text)

    groupnumbers3 = re.search('分.{0,10}?治疗组[和与]对照组.{0,10}?各(\d{1,3})[例名位个人]',text)

    groupnumbers4 = re.search('分.{0,10}?对照组[和与]治疗组.{0,10}?各(\d{1,3})[例名位个人]',text)

    Groupnumber = ''

    if groupnumbers1 != None:

        Groupnumber = groupnumbers1.group(1)

    elif groupnumbers2 != None:

        Groupnumber = groupnumbers2.group(1)

    elif groupnumbers3 != None:

        Groupnumber = groupnumbers3.group(1)

    elif groupnumbers4 != None:

        Groupnumber = groupnumbers4.group(1)

    return Groupnumber

def getsexnumber(text):

    text = re.sub('学','',text)

    sexnumber1 = re.search('在.{0,100}?接受治疗的{0，1}(\d{2,4})[例名人个位]',text)

    sexnumber2 = re.search('纳入.{0,100}?(\d{2,4})[例名人个位]',text)

    sexnumber3 = re.search('共{0,1}收[集治].{0,100}?(\d{2,4})[例名人个位]',text)

    sexnumber4 = re.search('选[择取].{0,100}?(\d{2,4})[例名人个位]',text)

    sexnumber5 = re.search('全部.{0,100}?(\d{2,4})[例名人个位]均{0,1}',text)

    sexnumber6 = re.search('方法.{0,100}?(\d{2,4})[例名人个位]',text)

    sexnumber7 = re.search('(\d{2,4})[例名人个位]均为.{0,100}?患者',text)

    sexnumber8 = re.search('资料.{0,100}?(\d{2,4})[例名人个位]',text)

    sexnumber9 = re.search('患[者儿].{0,100}?(\d{2,4})[例名人个位]',text)

    sexnumber = ''

    if sexnumber1 != None:

        sexnumber = sexnumber1.group(1)

    elif sexnumber2 != None:

        sexnumber = sexnumber2.group(1)

    elif sexnumber3 != None:

        sexnumber = sexnumber3.group(1)

    elif sexnumber4 != None:

        sexnumber = sexnumber4.group(1)

    elif sexnumber5 != None:

        sexnumber = sexnumber5.group(1)

    elif sexnumber6 != None:

        sexnumber = sexnumber6.group(1)

    elif sexnumber7 != None:

        sexnumber = sexnumber7.group(1)

    elif sexnumber8 != None:

        sexnumber = sexnumber8.group(1)

    elif sexnumber9 != None:

        sexnumber = sexnumber9.group(1)

return sexnumber

**5. Age (including: age_rnghigh, age_rnglow, age_mean and age_sd) pattern rules and codes**

def getAgenumbers(text):

    text = re.sub('为|者','',text)

    text = re.sub('\s','',text)

    agenumber1 = re.search('(治疗组|对照组).+?(年龄\d{1,2}个{0,1}[月年]{0,1}[至~〜\-]\d{1,2}[月岁]{0,1}).{0,5}?(平均[个月至【】\d\.~〜\-\+±土士\(\)（）]{1,17}[月岁]{0,1}).+?(治疗组|对照组).+?(年龄\d{1,2}个{0,1}[月年]{0,1}[至~〜\-]\d{1,2}[月岁]{0,1}).{0,5}?(平均[个月至【】\d\.~〜\-\+±土士\(\)（）]{1,17}[月岁]{0,1})',text)

    agenumber2 = re.search('(治疗组|对照组).+?(年龄最[大小]\d{1,2}个{0,1}[月岁]{0,1}).{0,5}(年{0,1}龄{0,1}最[大小]\d{1,2}个{0,1}[月岁]{0,1}).{0,5}?(平均年{0,1}龄{0,1}[个月至【】\d\.~〜\-\+±土士\(\)（）]{1,17}[月岁]{0,1}).+?(治疗组|对照组).{0,30}?(年龄最[大小]\d{1,2}个{0,1}[月岁]{0,1}).{0,5}(年{0,1}龄{0,1}最[大小]\d{1,2}个{0,1}[月岁]{0,1}).{0,5}?(平均年{0,1}龄{0,1}[个月至【】\d\.~〜\-\+±土士\(\)（）]{1,17}[月岁]{0,1})',text)

    agenumber3 = re.search('(治疗组|对照组).+?(年龄最[大小]\d{1,2}个{0,1}[月岁]{0,1}).{0,5}(年{0,1}龄{0,1}最[大小]\d{1,2}个{0,1}[月岁]{0,1}).+?(治疗组|对照组).{0,30}?(年龄最[大小]\d{1,2}个{0,1}[月岁]{0,1}).{0,5}(年{0,1}龄{0,1}最[大小]\d{1,2}个{0,1}[月岁]{0,1})',text)

    agenumber4 = re.search('(治疗组|对照组).+?(年龄范围在[个月至【】\d\.~〜\-\+±土士\(\)（）]{1,15}[月岁]{0,1}).{0,5}?(平均年{0,1}龄{0,1}[个月至【】\d\.~〜\-\+±土士\(\)（）]{1,13}[月岁]{0,1}).+?(治疗组|对照组).+?(年龄范围在[个月至【】\d\.~〜\-\+±土士\(\)（）]{1,13}[月岁]{0,1}).{0,5}?(平均年{0,1}龄{0,1}[个月至【】\d\.~〜\-\+±土士\(\)（）]{1,15}[月岁]{0,1})',text)

    agenumber5 = re.search('(治疗组|对照组).+?(年龄分布在{0,1}[个月至【】\d\.~〜\-\+±土士\(\)（）]{1,17}[月岁]{0,1}).{0,5}?(平均年{0,1}龄{0,1}[个月至【】\d\.~〜\-\+±土士\(\)（）]{1,17}岁{0,1}).+?(治疗组|对照组).+?(年龄分布在{0,1}[个月至【】\d\.~〜\-\+±土士\(\)（）]{1,17}[月岁]{0,1}).{0,5}?(平均年{0,1}龄{0,1}[个月至【】\d\.~〜\-\+±土士\(\)（）]{1,17}[月岁]{0,1})',text)

    agenumber6 = re.search('(治疗组|对照组).+?(年龄分布在{0,1}[个月至【】\d\.~〜\-\+±土士\(\)（）]{1,17}[月岁]{0,1}).+?(治疗组|对照组).+?(年龄分布在{0,1}[个月至【】\d\.~〜\-\+±土士\(\)（）]{1,17}[月岁]{0,1})',text)

    agenumber7 = re.search('(治疗组|对照组).+?(年龄[个月至【】\d\.~〜\-\+±土士\(\)（）]{1,17}[月岁]{0,1}).{0,5}?(均值[个月至【】\d\.~〜\-\+±土士\(\)]{1,17}[月岁]{0,1}).+?(治疗组|对照组).+?(年龄[个月至【】\d\.~〜\-\+±土士\(\)（）]{1,17}[月岁]{0,1}).{0,5}?(均值[个月至【】\d\.~〜\-\+±土士\(\)（）]{1,17}[月岁]{0,1})',text)

    agenumber8 = re.search('(治疗组|对照组).+?(年龄[个月至【】\d\.~〜\-\+±土士\(\)（）]{1,17}[月岁]{0,1}).{0,5}?(平均年{0,1}龄{0,1}[个月至【】\d\.~〜\-\+±土士\(\)]{1,17}[月岁]{0,1}).+?(治疗组|对照组).+?(年龄[个月至【】\d\.~〜\-\+±土士\(\)（）]{1,17}[月岁]{0,1}).{0,5}?(平均年{0,1}龄{0,1}[个月至【】\d\.~〜\-\+±土士\(\)（）]{1,17}[月岁]{0,1})',text)

    agenumber9 = re.search('(治疗组|对照组).+?(平均年龄[个月至【】\d\.~〜\-\+±土士\(\)（）]{1,17}[月岁]{0,1}).+?(治疗组|对照组).+?(平均年龄[个月至【】\d\.~〜\-\+±土士\(\)（）]{1,17}[月岁]{0,1})',text)

    agenumber10 = re.search('(治疗组|对照组).+?(年龄[个月至【】\d\.~〜\-\+±土士\(\)（）]{1,17}[月岁]{0,1}).+?(治疗组|对照组).+?(年龄[个月至【】\d\.~〜\-\+±土士\(\)（）]{1,17}[月岁]{0,1})',text)

    agenumber = ''

    if agenumber1 != None:

        agenumber = agenumber1.group(1) + '  ' + agenumber1.group(2) + '  ' + agenumber1.group(3)+ '  ' + agenumber1.group(4)+ '  ' + agenumber1.group(5)+ '  ' + agenumber1.group(6)

    elif agenumber2 != None:

        agenumber = agenumber2.group(1) + '  ' + agenumber2.group(2) + '  ' + agenumber2.group(3)+ '  ' + agenumber2.group(4)+ '  ' + agenumber2.group(5)+ '  ' + agenumber2.group(6)+ '  ' + agenumber2.group(7)+ '  ' + agenumber2.group(8)

    elif agenumber3 != None:

        agenumber = agenumber3.group(1) + '  ' + agenumber3.group(2) + '  ' + agenumber3.group(3)+ '  ' + agenumber3.group(4)+ '  ' + agenumber3.group(5)+ '  ' + agenumber3.group(6)

    elif agenumber4 != None:

        agenumber = agenumber4.group(1) + '  ' + agenumber4.group(2) + '  ' + agenumber4.group(3)+ '  ' + agenumber4.group(4)+ '  ' + agenumber4.group(5)+ '  ' + agenumber4.group(6)

    elif agenumber5 != None:

        agenumber = agenumber5.group(1) + '  ' + agenumber5.group(2) + '  ' + agenumber5.group(3)+ '  ' + agenumber5.group(4)+ '  ' + agenumber5.group(5)+ '  ' + agenumber5.group(6)

    elif agenumber6 != None:

        agenumber = agenumber6.group(1) + '  ' + agenumber6.group(2) + '  ' + agenumber6.group(3)+ '  ' + agenumber6.group(4)

    elif agenumber7 != None:

        agenumber = agenumber7.group(1) + '  ' + agenumber7.group(2) + '  ' + agenumber7.group(3)+ '  ' + agenumber7.group(4)+ '  ' + agenumber7.group(5)+ '  ' + agenumber7.group(6)

    elif agenumber8 != None:

        agenumber = agenumber8.group(1) + '  ' + agenumber8.group(2) + '  ' + agenumber8.group(3)+ '  ' + agenumber8.group(4)+ '  ' + agenumber8.group(5)+ '  ' + agenumber8.group(6)

    elif agenumber9 != None:

        agenumber = agenumber9.group(1) + '  ' + agenumber9.group(2) + '  ' + agenumber9.group(3)+ '  ' + agenumber9.group(4)

    elif agenumber10 != None:

        agenumber = agenumber10.group(1) + '  ' + agenumber10.group(2) + '  ' + agenumber10.group(3)+ '  ' + agenumber10.group(4)

    agenumber = re.sub('\,|\:|，|。','',agenumber)

    return agenumber

def getAgenumber(text):

    text = re.sub('\s','',text)

    text = re.sub('为','',text)

    agenumbers1 = re.search('年龄[个月至【】\d\.~〜\-\+±土士\(\)（）]{1,17}[月岁]{0,1}.{0,5}?平均年{0,1}龄{0,1}[个月至【】\d\.~〜\-\+±土士\(\)（）]{1,17}[月岁]{0,1}',text)

    agenumbers2 = re.search('最[大小]年龄[个月至【】\d\.~〜\-\+±土士\(\)]{1,17}[月岁]{0,1}.{0,5}?最[大小]年龄[个月至【】\d\.~〜\-\+±土士\(\)]{1,17}[月岁]{0,1}.{0,5}?平均年{0,1}龄{0,1}[个月至【】\d\.~〜\-\+±土士\(\)]{1,17}[月岁]{0,1}',text)

    agenumbers3 = re.search('平均年龄[个月至【】\d\.~〜\-\+±土士\(\)（）a-zA-Z]{1,17}[月岁]{0,1}',text)

    agenumbers4 = re.search('年龄自{0,1}[【】\d\.~〜\-\+±土士\(\)（）月个至]{1,17}[月岁]{0,1}',text)

    agenumbers = ''

    if agenumbers1 != None:

        agenumbers = agenumbers1.group()

    elif agenumbers2 != None:

        agenumbers = agenumbers2.group()

    elif agenumbers3 != None:

        agenumbers = agenumbers3.group()

    elif agenumbers4 != None:

        agenumbers = agenumbers4.group()

    return agenumbers

def getgroupKind(text):

    groupKind1 = re.search('(年龄为{0,1}\d{1,2}个{0,1}[月年]{0,1}[至~〜\-]\d{1,2}[月岁]{0,1}).{0,5}?(平均年{0,1}龄{0,1}为{0,1}[个月至【】\d\.~〜\-\+±土士\(\)（）]{1,17}[月岁]{0,1})',text)

    groupKind2 = re.search('(年龄最[大小]\d{1,2}岁{0,1}).{0,5}?(年{0,1}龄{0,1}最[大小]\d{1,2}[月岁]{0,1}).{0,5}?(平均年{0,1}龄{0,1}[个月至【】\d\.~〜\-\+±土士\(\)（）]{1,17}[月岁]{0,1})',text)

    groupKind3 = re.search('(年龄最[大小]\d{1,2}岁{0,1}).{0,5}?(年{0,1}龄{0,1}最[大小]\d{1,2}[月岁]{0,1})',text)

    groupKind4 = re.search('(年龄范围在[个月至【】\d\.~〜\-\+±土士\(\)（）]{1,17}[月岁]{0,1}).{0,5}?(平均年{0,1}龄{0,1}[个月至【】\d\.~〜\-\+±土士\(\)（）]{1,17}[月岁]{0,1})',text)

    groupKind5 = re.search('(年龄分{0,1}布{0,1}在{0,1}[个月至【】\d\.~〜\-\+±土士\(\)（）]{1,17}[月岁]{0,1}).{0,5}?(平均年{0,1}龄{0,1}[个月至【】\d\.~〜\-\+±土士\(\)（）]{1,17}[月岁]{0,1})',text)

    groupKind6 = re.search('(年龄分{0,1}布{0,1}在{0,1}[个月至【】\d\.~〜\-\+±土士\(\)（）]{1,17}[月岁]{0,1})',text)

    groupKind7 = re.search('(年龄[个月至【】\d\.~〜\-\+±土士\(\)（）]{1,17}[月岁]{0,1}).{0,5}?(均值[个月至【】\d\.~〜\-\+±土士\(\)（）]{1,17}[月岁]{0,1})',text)

    groupKind8 = re.search('(年龄[个月至【】\d\.~〜\-\+±土士\(\)（）]{1,17}[月岁]{0,1}).{0,5}?(平均年{0,1}龄{0,1}[个月至【】\d\.~〜\-\+±土士\(\)（）]{1,17}[月岁]{0,1})',text)

    groupKind9 = re.search('(平均年{0,1}龄{0,1}[个月至【】\d\.~〜\-\+±土士\(\)（）]{1,17}[月岁]{0,1})',text)

    groupKind10 = re.search('(年龄[个月至【】\d\.~〜\-\+±土士\(\)（）]{1,17}[月岁]{0,1})',text)

    groupKind = ''

    if groupKind1 != None:

        groupKind = groupKind1.group(1)+'  '+groupKind1.group(2)

    elif groupKind2 != None:

        groupKind = groupKind2.group(1)+'  '+groupKind2.group(2)+'  '+groupKind2.group(3)

    elif groupKind3 != None:

        groupKind = groupKind3.group(1)+'  '+groupKind3.group(2)

    elif groupKind4 != None:

        groupKind = groupKind4.group(1)+'  '+groupKind4.group(2)

    elif groupKind5 != None:

        groupKind = groupKind5.group(1)+'  '+groupKind5.group(2)

    elif groupKind6 != None:

        groupKind = groupKind6.group(1)

    elif groupKind7 != None:

        groupKind = groupKind7.group(1)+'  '+groupKind7.group(2)

    elif groupKind8 != None:

        groupKind = groupKind8.group(1)+'  '+groupKind8.group(2)

    elif groupKind9 != None:

        groupKind = groupKind9.group(1)

    elif groupKind10 != None:

        groupKind = groupKind10.group(1)

    return groupKind

def getageMax(text):

    ageMax1 = re.search('年龄最大(\d{1,4}[月岁]{0,1})',text)

    ageMax2 = re.search('最大年龄(\d{1,4}[月岁]{0,1})',text)

    ageMax3 = re.search('最大(\d{1,4}[月岁]{0,1})',text)

    ageMax4 = re.search('[至~〜\-](\d{1,2}[月岁]{0,1})',text)

    ageMax = ''

    if ageMax1 != None:

        ageMax = ageMax1.group(1)

    elif ageMax2 != None:

        ageMax = ageMax2.group(1)

    elif ageMax3 != None:

        ageMax = ageMax3.group(1)

    elif ageMax4 != None:

        ageMax = ageMax4.group(1)

    if re.search('[\u4e00-\u9fa5]',ageMax) == None:

        ageMax = ageMax + '岁'

    return ageMax

def getageMin(text):

    ageMin1 = re.search('年龄最小(\d{1,4}[月岁]{0,1})',text)

    ageMin2 = re.search('最小年龄(\d{1,4}[月岁]{0,1})',text)

    ageMin3 = re.search('最小(\d{1,4}[月岁]{0,1})',text)

    ageMin4 = re.search('(\d{1,2}个{0,1}[月岁]{0,1})[至~\-〜]',text)

    ageMin = ''

    if ageMin1 != None:

        ageMin = ageMin1.group(1)

    elif ageMin2 != None:

        ageMin = ageMin2.group(1)

    elif ageMin3 != None:

        ageMin = ageMin3.group(1)

    elif ageMin4 != None:

        ageMin = ageMin4.group(1)

    if re.search('[\u4e00-\u9fa5]',ageMin) == None:

        ageMin = ageMin + '岁'

    return ageMin

def getageMean(text):

    ageMean1 = re.search('年龄\({0,1}(\d{0,3}\.{0,1}\d{0,3})[\(\+±土士]',text)

    ageMean2 = re.search('平均年龄\({0,1}(\d{0,3}\.{0,1}\d{0,3})[\(\+±土士]',text)

    ageMean3 = re.search('平均\({0,1}(\d{0,3}\.{0,1}\d{0,3})[\(\+±土士]',text)

    ageMean4 = re.search('平均(\d{0,3}\.{0,1}\d{0,3}[月岁]{0,1})',text)

    ageMean5 = re.search('平均年龄(\d{0,3}\.{0,1}\d{0,3}[月岁]{0,1})',text)

    ageMean6 = re.search('均值\({0,1}(\d{0,3}\.{0,1}\d{0,3})[\(\+±土士]',text)

    ageMean = ''

    if ageMean1 != None:

        ageMean = ageMean1.group(1)

    elif ageMean2 != None:

        ageMean = ageMean2.group(1)

    elif ageMean3 != None:

        ageMean = ageMean3.group(1)

    elif ageMean4 != None:

        ageMean = ageMean4.group(1)

    elif ageMean5 != None:

        ageMean = ageMean5.group(1)

    elif ageMean6 != None:

        ageMean = ageMean6.group(1)

    if re.search('[\u4e00-\u9fa5]',ageMean) == None:

        ageMean = ageMean + '岁

    return ageMean

def getageStandard(text):

    ageStandard1 = re.search('年龄\({0,1}\d{0,3}\.{0,1}\d{0,3}[\(\+±土士](\d{0,3}\.{0,1}\d{0,3}\){0,1}[月岁]{0,1})',text)

    ageStandard2 = re.search('平均年龄\({0,1}\d{0,3}\.{0,1}\d{0,3}[\(\+±土士](\d{0,3}\.{0,1}\d{0,3}\){0,1}[月岁]{0,1})',text)

    ageStandard3 = re.search('均值\({0,1}\d{0,3}\.{0,1}\d{0,3}[\(\+±土士](\d{0,3}\.{0,1}\d{0,3}\){0,1}[月岁]{0,1})',text)

    ageStandard4 = re.search('平均\({0,1}\d{0,3}\.{0,1}\d{0,3}[\(\+±土士](\d{0,3}\.{0,1}\d{0,3}\){0,1}[月岁]{0,1})',text)

    ageStandard = ''

    if ageStandard1 != None:

        ageStandard = ageStandard1.group(1)

    elif ageStandard2 != None:

        ageStandard = ageStandard2.group(1)

    elif ageStandard3 != None:

        ageStandard = ageStandard3.group(1)

    elif ageStandard4 != None:

        ageStandard = ageStandard4.group(1)

    ageStandard = re.sub('平|均|年|龄|\d{0,3}\.{0,1}\d{0,3}±|\d{0,3}\.{0,1}\d{0,3}土|\d{0,3}\.{0,1}\d{0,3}士|\(|\)|\+','',ageStandard)

    if re.search('[\u4e00-\u9fa5]',ageStandard) == None:

        ageStandard = ageStandard +'岁'

    return ageStandard
